# Association between weight-adjusted-waist index and retinopathy among American adults: a cross-sectional study and mediation analysis

**DOI:** 10.3389/fnut.2025.1556065

**Published:** 2025-07-01

**Authors:** Junmeng Li, Qianshuo Yin, Jianchen Hao, Ruilin Zhu, Jing Zhang, Yadi Zhang, Xiaopeng Gu, Zihui Wu, Liu Yang

**Affiliations:** Department of Ophthalmology, Peking University First Hospital, Beijing, China

**Keywords:** WWI, retinopathy, HbA1c, NHANES (National Health and Nutrition Examination Survey), mediation analysis

## Abstract

**Background:**

Retinopathy, a microvascular complication linked to obesity and metabolic dysfunction, is characterized by the absence of reliable anthropometric predictors. The weight-adjusted waist index (WWI), incorporating waist circumference and weight, may better capture visceral adiposity and its pathological impact. This study investigates the association between WWI and retinopathy in U.S. adults and explores the potential mediating role of glycated hemoglobin (HbA1c).

**Methods:**

A cross-sectional analysis was conducted on 5,572 adults aged ≥40 years from the 2005–2008 National Health and Nutrition Examination Survey (NHANES), in which retinopathy was assessed using retinal imaging. Multivariate logistic regression, restricted cubic spline models, subgroup analyses, and threshold effect analysis were used to evaluate the WWI-retinopathy relationship. Receiver operating characteristic curves (ROC) were employed to compare the predictive ability of WWI to other obesity indices. Mediation analysis assessed the role of HbA1c.

**Results:**

Of 5,572 participants, 683 had retinopathy. Each unit increase in WWI was associated with a 31% higher risk of retinopathy (OR = 1.31, 95% CI: 1.16–1.48), increasing to 87% (OR = 1.87) in the highest quintile. WWI showed a dose-response relationship with retinopathy severity and consistent associations across subgroups. WWI outperformed weight, body mass index (BMI), and abdominal body shape index (ABSI) in predicting retinopathy (*P* < 0.05). Mediation analysis revealed that 30.02% of the WWI-retinopathy association was mediated by HbA1c (*P* < 0.001).

**Conclusion:**

WWI serves as a stronger predictor of retinopathy than conventional obesity measures. The partial mediation by HbA1c highlights the interconnected roles of visceral fat and hyperglycemia in retinal microvascular damage.

## 1 Introduction

Retinopathy is characterized in various studies by the presence of microaneurysms, retinal hemorrhages, hard exudates, cotton-wool spots, retinal venous abnormalities, intraretinal microvascular abnormalities, and neovascularization (1, 2). Among these, diabetic retinopathy (DR) and hypertensive retinopathy are the most prevalent forms and represent leading causes of vision loss and blindness in adults (3, 4). While hyperglycemia is widely recognized as a primary driver of DR, accumulating evidence suggests that central obesity, insulin resistance, and chronic low-grade inflammation are associated with retinopathy lesions in both non-diabetic and diabetic patients (5, 6).

Obesity has become a major global public health challenge, with its prevalence increasing substantially over recent decades (7). Projections indicate that by 2030, nearly half of the adult population in the United States will meet the diagnostic criteria for obesity (8). Central obesity and the excessive adipose tissue accumulation observed in obese individuals play a significant role in the development of insulin resistance syndrome and cardiovascular disease (9–11). The occurrence of retinal lesions is closely associated with the inflammatory cascade triggered by obesity and dysregulated fat metabolism. In obesity, immune cells infiltrating areas of excessive adipose tissue release pro-inflammatory cytokines such as IL-1β and IL-6, which disrupt insulin signaling and glucose homeostasis (12–16). These inflammatory mediators not only contribute to the development of insulin resistance but also induce microvascular damage, ultimately facilitating the onset of complications such as retinopathy (5, 16, 17).

Currently, the most widely used international indicator for assessing obesity is the body mass index (BMI). However, BMI lacks the ability to differentiate between fat and muscle mass, which imposes certain limitations. A meta-analysis comprising 25 studies from 12 countries revealed that approximately half of individuals with a normal BMI actually present with excess body fat (18). Furthermore, some studies have indicated no significant association between BMI and retinopathy, suggesting that BMI may not be an ideal predictor for retinopathy risk (1).

The recently introduced weight-adjusted waist index (WWI) combines waist circumference (WC) with the logarithmic transformation of body weight, effectively reducing the confounding effects of BMI and offering a more precise reflection of visceral fat accumulation (19, 20). Studies have shown that WWI is strongly associated with total abdominal fat mass, visceral fat, and subcutaneous fat, highlighting its considerable clinical utility in predicting the risk of cardiovascular diseases and type 2 diabetes mellitus (21, 22). Research indicates that each unit increase in WWI is independently associated with a significant 114%−196% higher risk of developing type 2 diabetes mellitus, independent of age, sex, lifestyle, or other cardiovascular risk factors (23, 24). Despite its considerable potential for predicting obesity-related diseases, the relationship between WWI and a broader spectrum of retinopathies remains underexplored.

Glycated hemoglobin (HbA1c) is a pivotal biomarker for assessing long-term glycemic control and is widely employed in both the diagnosis and management of diabetes. Emerging evidence suggests that HbA1c acts as a mediator in the relationship between dietary patterns and DR, implying that improving dietary habits and reducing HbA1c levels could potentially attenuate the risk of DR (25). However, the association between WWI and retinopathy in a broader context, as well as the potential mediating role of HbA1c in this relationship, remains inadequately understood and warrants further exploration.

Therefore, this study is the first to utilize cross-sectional data from the National Health and Nutrition Examination Survey (NHANES) 2005–2008 to investigate the association between WWI and retinopathy. It aims to evaluate the superior predictive ability of WWI compared to other obesity metrics for retinopathy and to explore the potential mediating role of HbA1c in the relationship between WWI and retinopathy. The findings of this study are expected to provide a critical foundation for the early identification of high-risk populations for retinopathy and to guide the development of more targeted preventive and intervention strategies, ultimately improving patients' visual function and quality of life.

## 2 Method

### 2.1 Data sources

Data from the 2005–2008 NHANES were analyzed. This survey represents a nationwide initiative conducted by the National Center for Health Statistics (NCHS) to assess the nutritional and health status of the U.S. population. A stratified, multistage probability sampling design was employed to create a nationally representative sample of non-institutionalized Americans (13). All research methods associated with NHANES were approved by the NCHS Research Ethics Review Board, and all participants provided written informed consent. Comprehensive details regarding the study design and data are publicly accessible on the official website (www.cdc.gov/nchs/nhanes/).

The inclusion criteria for NHANES participants were as follows: completion of retinal imaging and grading (as described below), along with provision of demographic information, including age, sex, race/ethnicity, educational attainment, and socioeconomic status.

### 2.2 Study population

**Figure 1:**
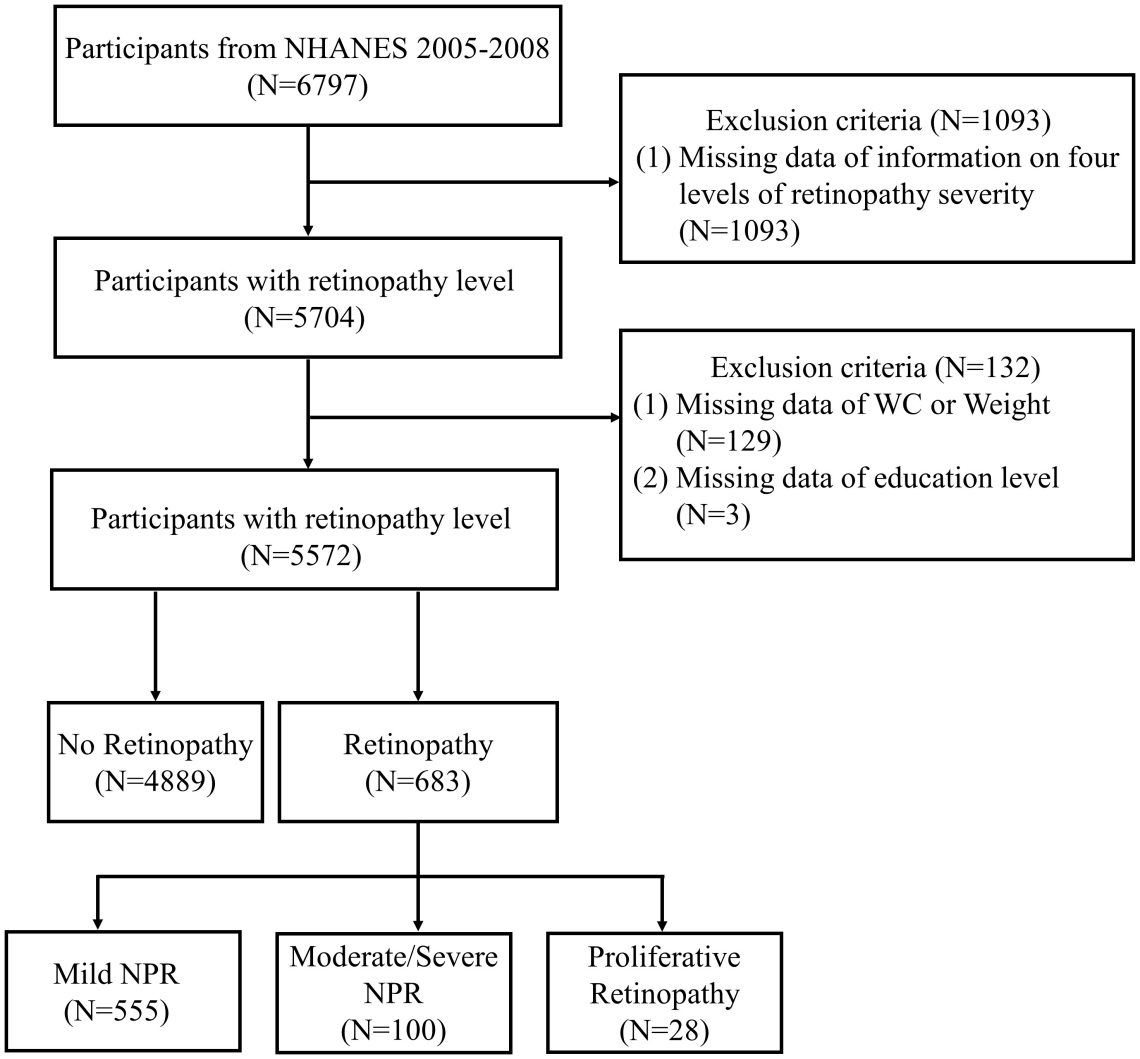
Flow chart of sample selection from NHANES 2005–2008. NHANES, National Health and Nutrition Examination Survey; WC, waist circumference; NPR, non-proliferative retinopathy.

Demographic, examination, and laboratory data from the NHANES 2005–2008 were analyzed, encompassing 6,797 participants aged 40 years or older. Participants with complete data on retinopathy level and the WWI were included in the analysis. According to Figure 1 in the flowchart, a total of 5,704 participants had available data on retinopathy severity grading. 126 participants with missing WC or weight data (and consequently WWI data) were excluded. Ultimately, 5,572 adults were retained for the final analysis.

### 2.3 Evaluation of retinopathy severity

Consistent with previous research, the identification and severity grading of retinopathy, that is the primary outcome of this study, relied on retinal image classification outcomes, rather than on self-reported DR data from questionnaires (26–29). Valluru et al. (30) explored the concordance between self-reported DR and retinal image-based DR classification, revealing a substantial mismatch. Studies showed that only 28.7% of patients who self-reported no DR and 49.8% of those who self-reported DR had concordant findings in retinal imaging. Gibson (31), Nwanyanwu (32), and their colleagues conducted studies on DR awareness, revealing that ~70% of DR patients were unaware of their diagnosis. Consequently, this study included both diabetic and non-diabetic participants in the No Retinopathy Group without further stratification according to self-reported diabetes or DR status. This approach mitigates potential biases arising from inaccuracies in self-reported data.

Visual health examinations conducted by NHANES between 2005 and 2008 included retinal imaging for participants aged 40 years and older. For each eye of the participants, two non-mydriatic images were captured using the Canon CR6-45NM ophthalmic imaging system in conjunction with the Canon EOS 10D digital camera (Canon USA, One Canon Park, Melville, New York). The Wisconsin team provided a detailed description of the image acquisition, grading, and quality control procedures. Retinal status classification and evaluation were performed utilizing the Early Treatment Diabetic Retinopathy Study (ETDRS) grading system, ensuring consistency and accuracy in diagnosing retinal conditions. The presence of any of the following conditions was defined as retinopathy: (i) hard exudates/soft exudates; (ii) intraretinal microvascular abnormalities; (iii) microaneurysms; (iv) hemorrhages; (v) non-proliferative or proliferative diabetic retinopathy, fibrous proliferation; or any evidence of diabetic retinopathy. Based on these criteria, participants were classified into either the retinopathy group (levels ≥14) or the non-retinopathy group (levels 10–13).

The No Retinopathy Group comprises cases characterized by the absence of retinopathy, questionable retinopathy, or non-diabetic retinal disease specific retinopathy. The levels of retinopathy severity can be further divided into (i) mild non-proliferative retinopathy (NPR): levels 14–31, microaneurysms only; (ii) moderate-severe NPR: levels 41–51, more than microaneurysms but less than proliferative retinopathy; (iii) proliferative retinopathy (PR): levels 60–80, evidence of neovascularization.

### 2.4 Evaluation of weight-adjusted-waist index

The primary predictive factor in this study is the WWI, an anthropometric measure based on WC and weight, employed to assess obesity. A higher WWI score indicates a greater degree of obesity. Trained health technicians measured WC and weight at the Mobile Examination Center (MEC). WWI is calculated by dividing waist circumference (cm) by the square root of weight (kg). Participants were grouped based on their WWI quartiles for subsequent analysis, with WWI treated as a continuous variable for the analysis. In this study, WWI was treated as the primary exposure factor. Alternative obesity indices, including WC, BMI, and abdominal body shape index (ABSI), were also employed as comparative measures of obesity exposure. Krakauer NY and Krakauer JC (33) define ABSI as the *WC* divided by *BMI*^2/3^ multiplied by *height*^1/2^. To address the issue of the ABSI regression coefficient being overly large, the ABSI value was multiplied by 1,000.

### 2.5 Evaluation of covariates of interest

Demographic information, including age, gender, race/ethnicity, educational attainment, and the ratio of family income to poverty (PIR), was collected via standardized questionnaires administered during structured family interviews. Systolic blood pressure (SBP), diastolic blood pressure (DBP), weight, WC, and BMI were measured by trained health technicians. Participants were instructed to fast for a minimum of 8 h prior to the collection of fasting blood samples, which were subsequently analyzed for high-density lipoprotein cholesterol (HDL-C, mg/dl) and total cholesterol (TCHOL, mg/dl).

### 2.6 Statistical analysis

We conducted *t*-tests for continuous variables and χ^2^ tests for categorical variables to analyze the differences in baseline characteristics between the Non-Retinopathy and Retinopathy groups. Continuous variables were expressed as mean ± standard deviation, whereas categorical variables were presented as frequency or percentage. To evaluate the association between WWI and the risk of retinopathy, we employed two unadjusted multivariate logistic regression models, calculating odds ratios (OR) and 95% confidence intervals (CI). The first model treated retinopathy as a binary outcome variable (presence or absence of retinopathy), In the first model, retinopathy was treated as a binary outcome variable (presence or absence of retinopathy), while in the second model, outcomes were stratified into different stages of retinopathy (non-retinopathy, mild NPR, and moderate-severe NPR/PR). In the second model, individuals exhibiting PR (*N* = 28) were aggregated with those demonstrating moderate-severe NPR due to limitations in sample size. The model was subsequently adjusted for covariates in a stepwise manner. Specifically, Model 1 included fundamental demographic variables such as gender, age, race, education level, and socioeconomic status. Model 2 further accounted for additional physiological parameters, including SBP, TCHOL, and HDL.

To assess the robustness, the continuous variable WWI was categorized into quintiles, and sensitivity analyses were conducted with the first quintile as a reference. Additionally, missing data for covariates were addressed using the random forest imputation method, which is well-suited for handling complex patterns of missingness. To investigate the potential non-linear relationship between WWI and retinopathy, restricted cubic splines (RCS) were employed, allowing for a more flexible modeling approach. This methodology ensures a thorough exploration of both linear and non-linear associations while maintaining model stability and interpretability.

Finally, we assessed the predictive ability of WWI and other obesity indicators for retinopathy using receiver operating characteristic (ROC) curves and the area under the curve (AUC) values. Subsequently, a mediation analysis was performed to evaluate the indirect, direct, and total effects of HbA1c as a mediator in the relationship between WWI and retinopathy. The mediation ratio was calculated as (indirect effect)/(indirect effect + direct effect) × 100%. The 95% CI was estimated using a bootstrap method with 1,000 resamples (34). The mediation analysis was conducted using the “mediation” package in R software (4.1.1). All statistical analyses were performed with R software (version 4.1.1) and EmpowerStats (version 4.1), and two-sided *P*-values < 0.05 were considered statistically significant.

## 3 Results

### 3.1 Demography

The study sample comprised 5,572 American adults aged 40 years and older. In comparison to adults without retinopathy, those with retinopathy (*N* = 683) were significantly older, more likely to be male and non-Hispanic Black, and exhibited lower education levels, PIR, DBP, TCHOL, and HDL, while having higher BMI, SBP, HbA1C, weight, WC, ABSI, and WWI (Table 1).

**Table 1:** Characteristics of U.S. participants stratified by retinopathy status in NHANES 2005–2008.

**Characteristic**	**No retinopathy *N* = 4,889**	**Retinopathy *N* = 683**	***P*-value**
**Age (year)**	**59.1** ±**12.4**	**62.1** ±**11.7**	<**0.001**
< 50	1,365 (27.9%)	117 (17.1%)	
≥50, < 60	1,165 (23.8%)	153 (22.4%)	
≥60, < 70	1,179 (24.1%)	222 (32.5%)	
≥70	1,180 (24.1%)	191 (28.0%)	
**Gender**			**0.006**
Male	2,415 (49.4%)	376 (55.1%)	
Female	2,474 (50.6%)	307 (44.9%)	
**Race**			<**0.001**
Mexican American	744 (15.2%)	125 (18.3%)	
Other Hispanic	342 (7.0%)	51 (7.5%)	
Non-Hispanic White	2,698 (55.2%)	283 (41.4%)	
Non-Hispanic Black	943 (19.3%)	205 (30.0%)	
Other race	162 (3.3%)	19 (2.8%)	
**Education**			<**0.001**
Less than 9th grade	658 (13.5%)	135 (19.8%)	
9th−12th grade	708 (14.5%)	130 (19.0%)	
High school graduate/GED	1,208 (24.7%)	172 (25.2%)	
Some college or AA degree	1,253 (25.6%)	164 (24.0%)	
College graduate or above	1,062 (21.7%)	82 (12.0%)	
**PIR**	**2.8** ±**1.6**	**2.5** ±**1.5**	<**0.001**
< 1.3	1,131 (23.1%)	176 (25.8%)	
≥1.3, < 5.0	2,720 (55.6%)	415 (60.8%)	
≥5.0	1,038 (21.2%)	92 (13.5%)	
**BMI (kg/m** ^2^ **)**	**29.1** ±**6.3**	**30.2** ±**6.3**	<**0.001**
< 25	1,315 (26.9%)	126 (18.4%)	
≥25, < 30	1,753 (35.9%)	262 (38.4%)	
≥30	1,821 (37.2%)	295 (43.2%)	
SBP (mmHg)	129.2 ± 19.4	136.7 ± 22.5	< 0.001
DBP (mmHg)	71.8 ± 13.2	70.6 ± 14.1	0.004
HbA1c (%)	5.7 ± 0.9	6.8 ± 1.8	< 0.001
TCHOL (mg/dl)	203.0 ± 41.1	196.8 ± 46.1	< 0.001
HDL (mg/dl)	53.8 ± 16.3	51.7 ± 14.9	0.007
Weight (kg)	81.7 ± 19.8	84.1 ± 19.5	0.001
WC (cm)	100.5 ± 15.1	103.9 ± 14.6	< 0.001
ABSI	82.6 ± 4.9	83.5 ± 4.6	< 0.001
WWI (1 cm/√kg)	11.2 ± 0.8	11.4 ± 0.8	< 0.001

NHANES, National Health and Nutrition Examination Survey; PIR, ratio of family income to poverty; BMI, body mass index; SBP, systolic blood pressure; DBP, diastolic blood pressure; HbA1c, glycated hemoglobin; TCHOL, total cholesterol; HDL, high-density lipoprotein; WC, waist circumference; ABSI, abdominal body shape index; WWI, weight-adjusted-waist index.

### 3.2 Relationship between WWI and retinopathy

The correlation between WWI and retinopathy indicates that a higher WWI is associated with an increased risk of retinopathy. Both the preliminary model and the adjusted model demonstrate a significant positive association between WWI and retinopathy. In the unadjusted model, WWI (OR: 1.42, 95% CI: 1.28–1.58, *P* < 0.001) and Q5 WWI were significant predictors of retinopathy (OR: 2.30, 95% CI: 1.76–2.99, *P* < 0.001). After adjusting for demographic characteristics such as age, gender, and race, Model 1 showed that the ORs for WWI and Q5 WWI were 1.41 (95% CI: 1.25–1.58, *P* < 0.001) and 2.24 (95% CI: 1.67–2.99, *P* < 0.001), respectively (Table 2).

**Table 2:** Association between WWI and retinopathy.

**Exposure**	**Non-adjusted**	**Adjust I**	**Adjust II**
WWI per 1 cm/√kg increase	1.42 (1.28, 1.58) ** < 0.001**	1.41 (1.25, 1.58) ** < 0.001**	1.31 (1.16, 1.48) ** < 0.001**
**Categorical**			
Quintile 1	Reference	Reference	Reference
Quintile 2	1.44 (1.08, 1.91) **0.013**	1.47 (1.10, 1.97) **0.009**	1.40 (1.04, 1.88) **0.026**
Quintile 3	1.42 (1.07, 1.89) **0.015**	1.42 (1.06, 1.90) **0.020**	1.31 (0.97, 1.77) 0.078
Quintile 4	1.78 (1.35, 2.34) ** < 0.001**	1.70 (1.27, 2.27) ** < 0.001**	1.50 (1.11, 2.02) **0.008**
Quintile 5	2.30 (1.76, 2.99) ** < 0.001**	2.24 (1.67, 2.99) ** < 0.001**	1.87 (1.38, 2.54) ** < 0.001**

Unsensitive analysis, converting the weight adjusted waist circumference index from a continuous variable to a categorical variable (quintile).

Non-adjusted model adjust for: none.

Adjust I model adjust for: gender; age; race.

Adjust II model adjust for: gender; age; race; education; PIR; SBP; TCHOL; HDL.

WWI, weight-adjusted-waist index; PIR, ratio of family income to poverty; SBP, systolic blood pressure; TCHOL, total cholesterol; HDL, high-density lipoprotein.

Bold values indicate statistically significant results (*P* < 0.05).

After full adjustment, participants with higher WWI units had a 30% higher risk of developing retinopathy (Model II: OR = 1.31, 95% CI: 1.16–1.48). Compared with participants in the lowest quintile of WWI, those in the highest quintile had an 87% higher risk of retinopathy (OR = 1.87, 95% CI: 1.38–2.54; *P* < 0.001; Table 2).

Compared to other predictors like Weight, WC, BMI, and ABSI, WWI maintains its predictive strength even after adjustments, particularly in *Z*-score standardization. WWI appears to be a more reliable and robust predictor (Table S1).

**Figure 2:**
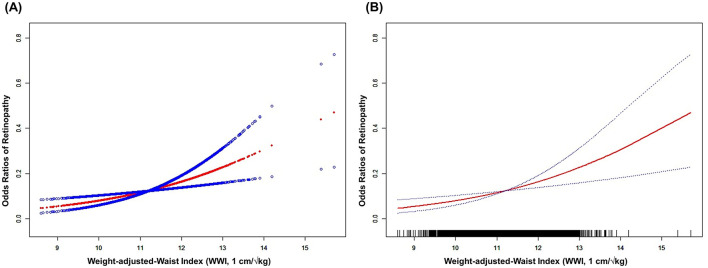
The association between WWI and retinopathy risk. **(A)** Scatterplot and fitted curve demonstrating a positive association between WWI and retinopathy risk, with a fitted regression curve (red line) and its 95% confidence interval (blue shaded area). **(B)** Smoothed curve highlights the smoothed trend (red line) between WWI and retinopathy risk, with dashed lines representing the 95% confidence interval. The rug plot at the bottom indicates the distribution of WWI values. The figure shows a non-linear relationship, with retinopathy risk increasing sharply at higher WWI thresholds. Age, Gender, Race, Education, PIR, SBP, TCHOL, and HDL were adjusted. WWI, weight-adjusted-waist index; PIR, ratio of family income to poverty; SBP, systolic blood pressure; TCHOL, total cholesterol; HDL, high-density lipoprotein.

Furthermore, the relationship between WWI and retinopathy risk was further analyzed using smooth curve fitting, which demonstrated a positive correlation (Figure 2).

### 3.3 Association between WWI and retinopathy severity

Patients with retinopathy (*N* = 683) were stratified into three groups according to retinal lesion severity: mild NPR (*N* = 555), moderate/severe NPR (*N* = 100), and PR (*N* = 28). The main predictive factor, WWI, along with various covariates, demonstrated significant variation across these three groups (Table S2).

**Figure 3:**
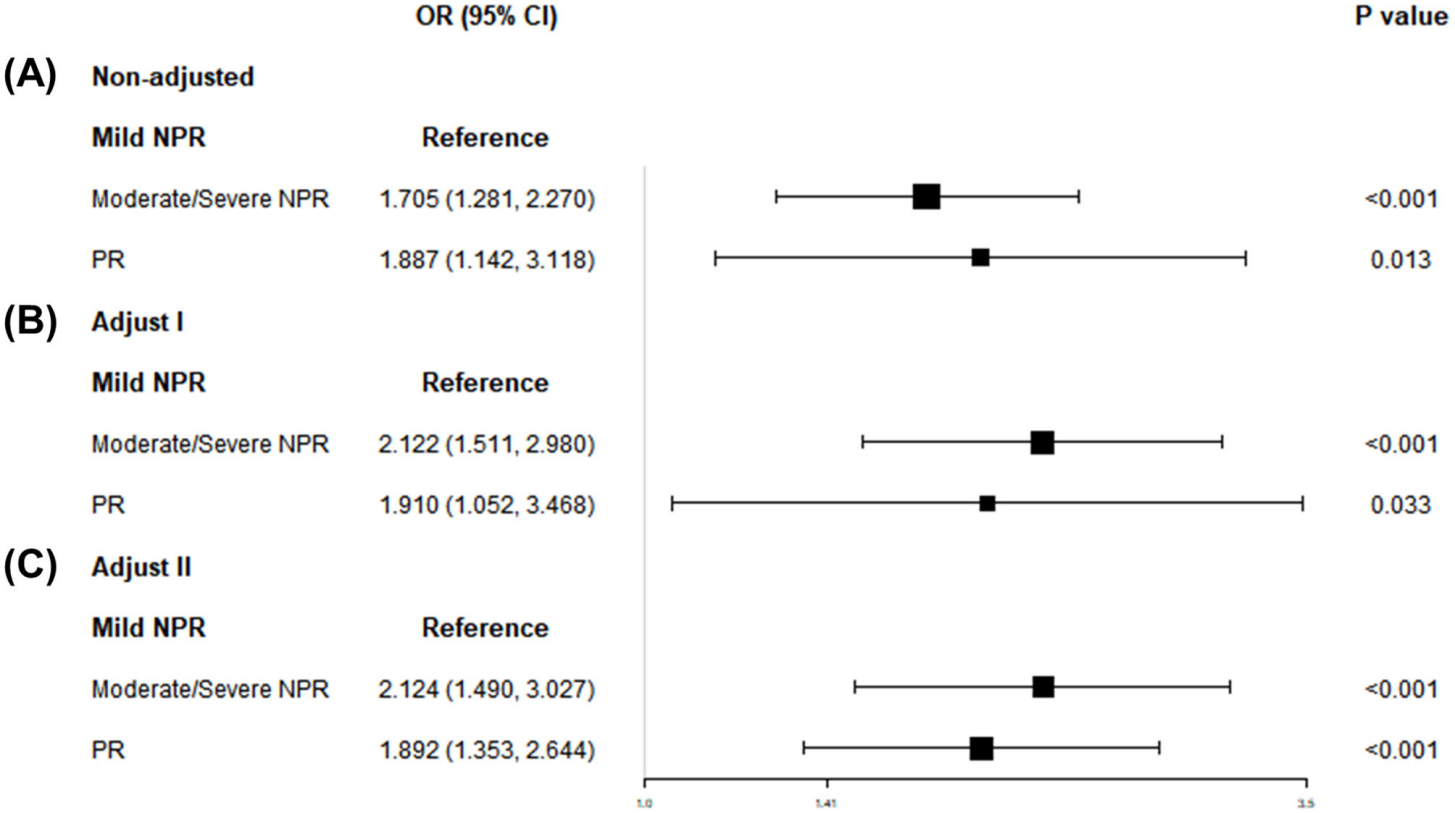
Associations of WWI with retinopathy severity, NHANES 2005–2008. **(A)** Model 1: non-adjusted. **(B)** Model 2: adjusted for age, sex, and race. **(C)** Model 3: adjusted for gender; age; race; education; PIR; SBP; TCHOL; HDL. WWI, weight-adjusted-waist index; NHANES, National Health and Nutrition Examination Survey; NPR, non-proliferative retinopathy; PR, proliferative retinopathy; PIR, ratio of family income to poverty; SBP, systolic blood pressure; DBP, diastolic blood pressure; TCHOL, total cholesterol; HDL, high-density lipoprotein; ABSI, abdominal body shape index.

Figure 3 depicts the association between WWI and retinopathy severity. After full adjustment, the likelihood of moderate/severe NPR and PR increased as WWI increased (OR: 2.12, 95% CI: 1.49–3.03; OR: 1.89, 95% CI: 1.35–2.64). Compared with participants in the lowest quintile of WWI, those in the highest quintile had significantly higher risks of moderate/severe NPR and PR (OR: 4.67, 95% CI: 2.05–10.65; OR: 11.75, 95% CI: 1.31–105.04; Table S3).

### 3.4 Subgroup analysis of the correlation between WWI and retinopathy

Subgroup analysis indicate that the positive correlation between WWI and retinopathy remained consistent across various subgroups, including age, gender, race, education, and PIR (all interaction *P*-values < 0.05), suggesting that the association between WWI and retinopathy is robust across diverse population groups. The positive correlation was observed consistently across subgroups. For instance, in the male subgroup, each additional unit of WWI was associated with a 21% increase in retinopathy risk (OR: 1.21, 95% CI: 1.02–1.43), and a similar significant association was observed in the female subgroup (OR: 1.40, 95% CI: 1.19–1.64; Table 3).

**Table 3:** Subgroups analyses of the effect of WWI on retinopathy.

**Subgroups**	***N***		**OR (95% CI) *P*-value**	***P* for interaction**
	**Total**	**Retinopathy**		
**Gender**				0.184
Male	2,791	376	1.21 (1.02, 1.43) 0.029	
Female	2,781	307	1.40 (1.19, 1.64) < 0.001	
**Age**				0.116
< 50	1,482	117	1.08 (0.82, 1.41) 0.595	
≥50, < 60	1,318	153	1.63 (1.27, 2.09) < 0.001	
≥60, < 70	1,401	222	1.20 (0.97, 1.49) 0.092	
≥70	1,371	191	1.35 (1.10, 1.66) 0.004	
**Race**				0.471
Mexican American	869	125	1.32 (1.00, 1.74) 0.053	
Other Hispanic	393	51	1.16 (0.75, 1.80) 0.509	
Non-Hispanic White	2,981	283	1.19 (1.00, 1.42) 0.056	
Non-Hispanic Black	1,148	205	1.50 (1.23, 1.84) < 0.001	
Other race	181	19	1.42 (0.72, 2.83) 0.313	
**Education**				0.368
Less than 9th grade	793	135	1.19 (0.92, 1.55) 0.181	
9th−12th grade	838	130	1.61 (1.25, 2.07) < 0.001	
High school graduate/GED	1,380	172	1.19 (0.95, 1.49) 0.140	
Some college or AA degree	1,417	164	1.35 (1.08, 1.70) 0.010	
College graduate or above	1,144	82	1.23 (0.91, 1.67) 0.178	
**PIR**				0.466
< 1.3	1,307	176	1.26 (1.02, 1.56) 0.033	
≥1.3, < 5.0	3,135	415	1.28 (1.10, 1.49) 0.001	
≥5.0	1,130	92	1.57 (1.14, 2.16) 0.006	

WWI, weight-adjusted-waist index; PIR, ratio of family income to poverty.

### 3.5 Assessing the diagnostic impact of various obesity parameters on retinopathy

Smooth curve fitting analysis revealed a segmented positive association between body weight, WC, BMI, ABSI, and retinopathy (Figure S1). Subsequently, a segmented regression model was utilized to determine the threshold effect (Table 4). Notably, breakpoints were identified in the relationship between body weight, WC, BMI, ABSI, and retinopathy. Below the threshold, a significant positive association was observed (*P* < 0.05), whereas above the threshold, this association became non-significant (*P* > 0.05).

**Table 4:** Threshold effect analysis of WWI, Weight, WC, BMI, and ABSI on retinopathy using a two-piecewise linear regression model.

**Parameter**	**WWI**	**Weight**	**WC**	**BMI**	**ABSI**
**Fitting by standard linear model**					
OR (95% CI)	1.31 (1.16, 1.48)	1.01 (1.00, 1.01)	1.01 (1.01, 1.02)	1.02 (1.01, 1.03)	1.02 (1.00, 1.04)
*P*-value	**< 0.001**	**0.022**	**< 0.001**	**0.002**	**0.026**
**Fitting by two-piecewise linear model**					
Breakpoint (*K*)	10.45	68	127.7	38.2	74.76
OR1 (< *K*)	1.97 (1.09, 3.58) **0.025**	1.03 (1.01, 1.05) **0.014**	1.01 (1.01, 1.02) ** < 0.001**	1.04 (1.02, 1.06) ** < 0.001**	1.34 (1.05, 1.72) **0.019**
OR2 (>*K*)	1.24 (1.08, 1.43) **0.003**	1.00 (1.00, 1.01) 0.341	0.98 (0.95, 1.01) 0.226	0.97 (0.93, 1.02) 0.214	1.01 (0.99, 1.03) 0.267
OR2/OR1	0.63 (0.33, 1.21) 0.163	0.97 (0.95, 1.00) 0.041	0.97(0.93, 1.00) 0.059	0.94 (0.89, 0.99) 0.019	0.75 (0.58, 0.97) 0.027
Logarithmic likelihood ratio test *P*-value	0.150	0.037	0.046	0.014	0.007

Age, gender, race, education, PIR, SBP, TCHOL, and HDL were adjusted.

Significant values are in bold.

WWI, weight-adjusted-waist index; WC, waist circumference; BMI, body mass index; ABSI, abdominal body shape index; SBP, systolic blood pressure; TCHOL, total cholesterol; HDL, high-density lipoprotein.

In the analysis of non-linear models, a consistent positive correlation between WWI and retinopathy was confirmed (Table 4). The fitted curve exhibits heightened sensitivity to changes in trends. WWI demonstrates robust discriminatory power across both the lower (low-risk) and higher (rapidly increasing risk) ranges of retinopathy risk. Compared with the breakpoints for weight, WC, BMI, and ABSI, the association between WWI and retinopathy risk was significantly stronger [WWI: OR1 (< *K*) 1.97; OR2 (>*K*) 1.24].

### 3.6 Mediation effect

**Figure 4:**
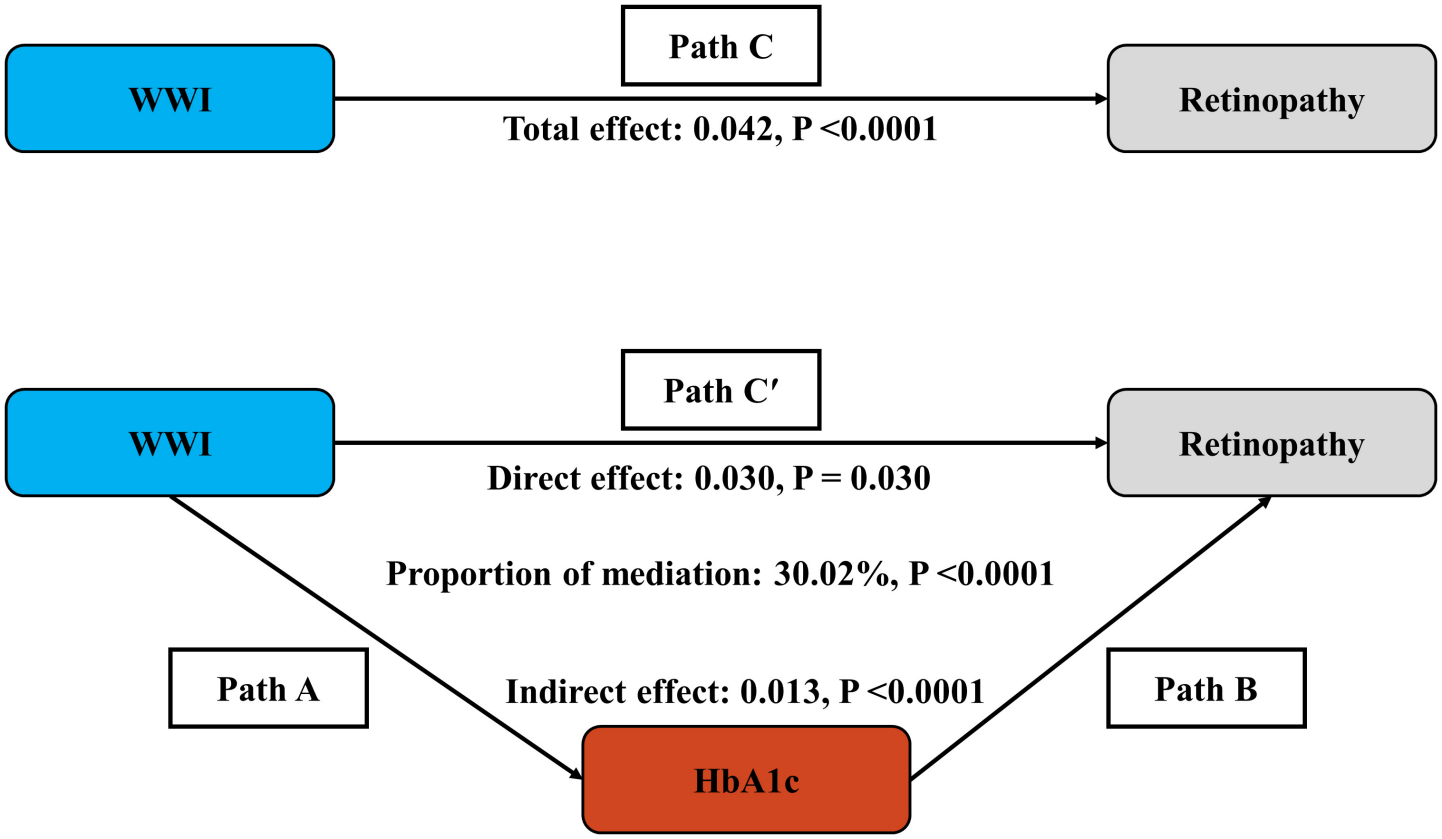
Schematic diagram of the mediation effect analysis. Path C indicates the total effect; path C′ indicates the direct effect. The indirect effect is estimated as the multiplication of paths A and B (path A*B). The mediated proportion is calculated as indirect effect/(indirect effect + direct effect) × 100%. Analyses were adjusted for Age, gender, race, education, PIR, SBP, TCHOL, and HDL. WWI, weight-adjusted-waist index; HbA1c, glycated hemoglobin; PIR, ratio of family income to poverty; SBP, systolic blood pressure; TCHOL, total cholesterol; HDL, high-density lipoprotein.

The mediation model presented in Figure 4 illustrates WWI as the independent variable, retinopathy as the dependent variable, and HbA1c as the mediator. As shown in Table S4, after adjusting for other covariates, there is a significant correlation between HbA1c and retinopathy (OR = 1.76, 95% CI: 1.65, 1.88), as well as between WWI and HbA1c (β = 0.23, 95% CI: 0.19, 0.27). Following the adjustment for all covariates, the mediating effect of HbA1c was evident, with an indirect effect of 0.013 (*P* < 0.0001) and a direct effect of 0.030 (*P* = 0.030). The mediation proportion was calculated at 30.02% (*P* < 0.0001).

### 3.7 ROC analysis for retinopathy

For diagnosing retinopathy, the AUC values for WWI, body weight, WC, BMI, and ABSI were 0.577, 0.538, 0.568, 0.556, and 0.548, respectively (Table S5). The results showed that WWI had a better predictive ability for retinopathy compared to Weight, BMI, and ABSI. WWI demonstrated the highest specificity (0.669) and accuracy (0.642), with moderate sensitivity (0.452). It is also worth noting that WWI had the lowest N-for-diagnose value (8.26). After gender stratification, the AUC for WWI remained higher than those of other obesity indicators in women for diagnosing retinopathy (Table S5).

WWI demonstrates higher AUC (0.579) compared to individual predictors like age (0.568) and gender (0.528), and it shows statistically significant improvements when combined with other factors (e.g., Race-WWI 0.623, education-WWI 0.605; *P* < 0.001), indicating its incremental value for retinopathy (Table S6).

## 4 Discussion

This study investigated the association between WWI and retinopathy in U.S. adults using the NHANES database. In a cohort of 5,572 participants aged 40 years and older, a higher WWI was associated with an increased likelihood of retinopathy. Subgroup analyses and interaction tests confirmed that this association remained consistent across diverse populations. Notably, the relationship between WWI and retinopathy showed a stronger association compared to other common obesity markers (e.g., weight, WC, BMI, and ABSI), indicating that WWI may be a more reliable predictor of retinopathy. Additionally, our results reveal the potential mediating role of HbA1c in this association, highlighting a pathway through which WWI may influence retinopathy risk.

### 4.1 The relationship between obesity and retinopathy

The robust association between WWI and retinopathy remained consistent across adjusted models, even after accounting for demographic confounders, socioeconomic factors, and metabolic covariates. Specifically, participants in the highest quintile of WWI exhibited an ~90% higher risk of developing retinopathy compared to those in the lowest quintile. This supports earlier studies suggesting that the risk of developing retinopathy is strongly associated with obesity (35, 36). The present study further investigated four conventional obesity indicators—body weight, WC, BMI and ABSI—in relation to retinopathy risk (Figure S1). Traditional metrics such as weight and BMI exhibited limited predictive capability as they fail to account for fat distribution and body composition (37, 38). However, WWI appears to be a more reliable predictor than conventional obesity metrics, especially after standardizing for *Z*-scores (Table S1). This is a pivotal observation, given that WWI integrates both weight and WC—factors that may more accurately reflect fat distribution, a key determinant of systemic inflammation and insulin resistance (14, 15).

Our study demonstrated that WWI also predicts retinopathy severity. Specifically, higher WWI was associated with an increased likelihood of both moderate-severe NPR and PR. These findings are consistent with prior research indicating that higher levels of abdominal obesity contribute to more severe forms of retinopathy (9). Subgroup analysis revealed that gender, age, race, education, and PIR had no impact on the positive association between WWI and retinopathy (interaction *P*-values >0.05), indicating that the positive association is stable across different population subgroups.

Our study also addressed the threshold effects of obesity parameters using segmented regression models. The threshold effects observed for weight, WC, BMI, and ABSI suggest diminishing predictive utility at extreme obesity levels, whereas WWI maintained a significant risk gradient. The results indicated that the association between WWI and retinopathy was most pronounced below breakpoint (*K* = 10.45), suggesting that early intervention in individuals with lower levels of obesity may help reduce the risk of retinopathy. This finding aligns with the growing body of literature emphasizing the importance of early detection and intervention in individuals with even modest levels of abdominal obesity (39–41).

### 4.2 ROC analysis for retinopathy

In this study, we used ROC analysis to compare the diagnostic performance of WWI, weight, WC, BMI, and ABSI for predicting retinopathy. WWI showed the highest AUC value (0.577) compared to Weight, BMI, and ABSI. WWI also demonstrated the highest specificity (0.669) and accuracy (0.642), suggesting that WWI has moderate but significant discriminatory power in identifying individuals at risk of retinopathy.

When performing gender stratification, WWI continued to show higher AUC values (0.615) in women compared to other obesity indices, reinforcing its diagnostic potential across different demographic groups. This finding is consistent with the growing recognition that abdominal obesity and its associated risks may vary between men and women (9). The higher diagnostic performance of WWI in women may be attributed to differences in fat distribution patterns between genders (42, 43).

The ROC analysis also demonstrated that combining WWI with demographic variables, resulted in significant improvements in AUC. For instance, the AUC for the interaction of race-WWI was 0.623, and for education-WWI, it was 0.605, both significantly higher than the AUCs for WWI alone. The ROC results further advocate for integrating WWI into multivariable screening tools, underscoring the incremental value of WWI in predicting retinopathy risk.

### 4.3 Exploring potential mechanisms linking WWI, HbA1c and retinopathy

The mediation analysis revealed that HbA1c accounted for 30% of the total effect of WWI on retinopathy, suggesting that hyperglycemia partially mediates this relationship. This finding helps connect adiposity-induced metabolic dysregulation with retinal pathology, as sustained hyperglycemia contributes to oxidative stress, endothelial dysfunction, and retinal microvascular occlusion.

Multiple studies have demonstrated that a higher WWI is a strong predictor of type 2 diabetes (T2DM) and its complications, including diabetic nephropathy, and outperforms conventional indicators such as BMI. This is particularly evident in younger individuals (< 60 years), reflecting WWI's sensitivity to visceral adiposity and related metabolic abnormalities. These findings provide further support for the mediating role of hyperglycemia in the WWI-retinopathy association.

Beyond hyperglycemia, adipose tissue dysfunction may also contribute to this relationship. WWI captures a state of increased visceral fat and muscle loss (sarcopenia), both of which are linked to insulin resistance—a key driver of diabetes-related complications. Dysfunctional adipose tissue reduces the secretion of anti-inflammatory adipokines such as adiponectin and increases pro-inflammatory cytokines like resistin, thereby promoting systemic inflammation and metabolic disturbance (10, 44–46).

Resistin, in particular, has been implicated in the progression of DR and is positively correlated with inflammatory markers such as C-reactive protein (CRP), a biomarker of vascular endothelial dysfunction (11, 47, 48). Moreover, visceral fat accumulation may further disturb glucose homeostasis through abnormal secretion of metabolic regulators like retinol-binding protein-1 and leptin, exacerbating hyperglycemia-induced retinal damage (10, 49). While these pathways offer biologically plausible mechanisms linking WWI to retinopathy, further research is required to elucidate the precise molecular and cellular processes involved.

### 4.4 Strengths and limitations

This study benefits from a nationally representative sample, rigorous adjustment for confounders, and advanced statistical methods (e.g., mediation analysis, non-linear modeling). However, the cross-sectional design precludes causal inference, and residual confounding (e.g., dietary patterns, physical activity) may persist. The small sample size for proliferative retinopathy (*N* = 28) limits precision in severity-stratified analyses, necessitating validation in larger cohorts. Additionally, retinal assessment methods (non-mydriatic photography vs. clinical grading) may lead to an underestimation of subtle lesions.

### 4.5 Future directions

Prospective studies should investigate whether WWI reduction through lifestyle or pharmacological interventions lowers incident retinopathy risk. Exploring other mediators (e.g., inflammatory biomarkers, endothelial dysfunction markers) could unravel additional mechanistic pathways. Finally, validating WWI's predictive performance in diverse ethnic populations and its integration into AI-based retinal screening algorithms warrants further research.

### 4.6 Conclusion

In conclusion, WWI emerges as a superior anthropometric indicator for retinopathy risk, transcending the limitations of conventional obesity metrics. Its association with retinopathy severity and partial mediation by HbA1c underscores the multifactorial nature of retinal microvascular disease, suggesting that interventions targeting glycemic control may help mitigate the effects of abdominal obesity on retinopathy development. However, additional longitudinal research is needed to establish the causal relationship underlying these observations.

## Data Availability

The original contributions presented in the study are included in the article/Supplementary material, further inquiries can be directed to the corresponding author.
