# Supplementary material for: Association between weight-adjusted-waist index and retinopathy among American adults: a cross-sectional study and mediation analysis
Source: Front Nutr. 2025 Jul 1;12:1556065. doi: 10.3389/fnut.2025.1556065 (PMC12259439; doi:10.3389/fnut.2025.1556065)
Supplement: Supplementary file 1 [file Table_1.docx]

**Supplementary Materials**

**Table S1.** Association between different obesity-related indices and retinopathy.

**Table S2.** Characteristics of retinopathy patients grouped by severity, NHANES 2005-2008.

**Table S3.** Association between WWI and retinopathy severity.

**Table S4.** The correlation between independent variable (WWI), dependent variable (retinopathy), and mediator variable (HbA1c).

**Table S5.** Comparison of the ability of different obesity-related indices to predict retinopathy**.**

**Table S6.** Comparison of the ability of WWI and different fundamental indices to predict retinopathy.

**Figure S1.** Smooth curve fitting for weight, WC, BMI, and ABSI with retinopathy risk.

**Table S1 Association between different obesity-related indices and retinopathy.**

| **Exposure** | **Non-adjusted** | **Adjust I** | **Adjust II** |
| --- | --- | --- | --- |
| WWI | **1.42 (1.28, 1.58)**  ***P* < 0.0001** | **1.41 (1.25, 1.58)**  ***P* < 0.0001** | **1.51 (1.18, 1.93)**  ***P* = 0.0010** |
| Weight | 1.01 (1.00, 1.01)  *P* = 0.0029 | - 1. (1.00, 1.01)   *P* = 0.0011 | - 1. (1.00, 1.01)   *P* = 0.0215 |
| WC | - 1. (1.01, 1.02)   *P* < 0.0001 | - 1. (1.01, 1.02)   *P* < 0.0001 | 1.01 (1.01, 1.02)  *P* = 0.0002 |
| BMI | - 1. (1.01, 1.04)   *P* < 0.0001 | - 1. (1.02, 1.04)   *P* < 0.0001 | - 1. (1.01, 1.03)   *P* = 0.0019 |
| ABSI | - 1. (1.02, 1.05)   *P* < 0.0001 | - 1. (1.01, 1.05)   *P* = 0.0041 | - 1. (1.00, 1.04)   *P* = 0.0261 |
| **Z-score Standardization** | | | |
| WWI Z score | **1.31 (1.21, 1.42)**  ***P* < 0.0001** | **1.30 (1.19, 1.43)**  ***P* < 0.0001** | **1.37 (1.14, 1.66)**  ***P* = 0.0010** |
| WEIGHT Z score | 1.12 (1.04, 1.21)  *P* = 0.0029 | 1.15 (1.06, 1.25)  *P* = 0.0011 | 1.11 (1.02, 1.21)  *P* = 0.0215 |
| WC Z score | 1.24 (1.15, 1.35)  *P* < 0.0001 | 1.23 (1.13, 1.33)  *P* < 0.0001 | 1.18 (1.08, 1.28)  *P* = 0.0002 |
| BMI Z score | 1.18 (1.09, 1.27)  *P* < 0.0001 | 1.20 (1.11, 1.30)  *P* < 0.0001 | 1.14 (1.05, 1.24)  *P* = 0.0019 |
| ABSI Z score | 1.20 (1.10, 1.30)  *P* < 0.0001 | 1.15 (1.04, 1.26)  *P* = 0.0041 | 1.12 (1.01, 1.23)  *P* = 0.0261 |

Non-adjusted model adjust for: None

Adjust I model adjust for: Gender; Age; Race

Adjust II model adjust for: Gender; Age; Race; Education; PIR; SBP; TCHOL; HDL.

Abbreviations: WWI, weight-adjusted-waist index; WC, waist circumference; BMI, body mass index; ABSI, abdominal body shape index; PIR, ratio of family income to poverty; SBP, systolic blood pressure; TCHOL, total cholesterol; HDL, high-density lipoprotein.

**Table S2 Characteristics of retinopathy patients grouped by severity, NHANES 2005-2008.**

| **Characteristic** | **Mild NPR** | **Moderate/Severe NPR** | **PR** | ***P* Value** |
| --- | --- | --- | --- | --- |
| **Gender** |  |  |  | 0.007 |
| Male | 315 (56.8%) | 50 (50.0%) | 11 (39.3%) |  |
| Female | 240 (43.2%) | 50 (50.0%) | 17 (60.7%) |  |
| **Age (year)** | 62.3 ± 12.0 | 60.7 ± 11.0 | 63.0 ± 7.1 | <0.001 |
| <50 | 97 (17.5%) | 19 (19.0%) | 1 (3.6%) |  |
| >=50, <60 | 126 (22.7%) | 22 (22.0%) | 5 (17.9%) |  |
| >=60, <70 | 165 (29.7%) | 37 (37.0%) | 20 (71.4%) |  |
| >=70 | 167 (30.1%) | 22 (22.0%) | 2 (7.1%) |  |
| **Race** |  |  |  | <0.001 |
| Mexican American | 93 (16.8%) | 26 (26.0%) | 6 (21.4%) |  |
| Other Hispanic | 43 (7.7%) | 3 (3.0%) | 5 (17.9%) |  |
| Non-Hispanic White | 253 (45.6%) | 25 (25.0%) | 5 (17.9%) |  |
| Non-Hispanic Black | 149 (26.8%) | 44 (44.0%) | 12 (42.9%) |  |
| Other race | 17 (3.1%) | 2 (2.0%) | 0 (0.0%) |  |
| **Education** |  |  |  | <0.001 |
| Less than 9th grade | 107 (19.3%) | 19 (19.0%) | 9 (32.1%) |  |
| 9th-12th grade | 99 (17.8%) | 25 (25.0%) | 6 (21.4%) |  |
| High School graduate/GED | 146 (26.3%) | 22 (22.0%) | 4 (14.3%) |  |
| Some college or AA degree | 137 (24.7%) | 20 (20.0%) | 7 (25.0%) |  |
| College graduate or above | 66 (11.9%) | 14 (14.0%) | 2 (7.1%) |  |
| **PIR** |  |  |  | <0.001 |
| <1.3 | 140 (25.2%) | 25 (25.0%) | 11 (39.3%) |  |
| >=1.3, <=3.5 | 270 (48.6%) | 47 (47.0%) | 15 (53.6%) |  |
| >3.5 | 145 (26.1%) | 28 (28.0%) | 2 (7.1%) |  |
| **SBP (mmHg)** | 135.8 ± 22.4 | 141.0 ± 23.3 | 140.4 ± 19.4 | <0.001 |
| **DBP (mmHg)** | 70.8 ± 14.3 | 70.6 ± 12.9 | 67.1 ± 15.6 | 0.091 |
| **HbA1c (%)** | 6.4 ± 1.5 | 8.3 ± 2.0 | 8.4 ± 1.9 | <0.001 |
| **TCHOL (mg/dL)** | 195.8 ± 46.1 | 199.7 ± 46.4 | 205.3 ± 45.9 | 0.431 |
| **HDL (mg/dL)** | 52.0 ± 14.7 | 50.4 ± 15.7 | 52.4 ± 16.3 | 0.722 |
| **Weight (kg)** | 83.1 ± 19.1 | 88.9 ± 22.0 | 85.7 ± 17.2 | <0.001 |
| **WC (cm)** | 102.8 ± 14.3 | 108.9 ± 15.3 | 107.7 ± 12.3 | <0.001 |
| **ABSI** | 83.3 ± 4.6 | 84.4 ± 4.9 | 84.8 ± 4.3 | <0.001 |
| **BMI (kg/m2)** | 29.8 ± 6.1 | 32.0 ± 7.0 | 31.3 ± 5.4 | <0.001 |
| <25.0 | 110 (19.8%) | 11 (11.0%) | 5 (17.9%) |  |
| >=25.0, <30.0 | 220 (39.6%) | 37 (37.0%) | 5 (17.9%) |  |
| >=30.0 | 225 (40.5%) | 52 (52.0%) | 18 (64.3%) |  |
| **WWI (****1 cm/√kg)** | 11.3 ± 0.7 | 11.6 ± 0.8 | 11.7 ± 0.7 | <0.001 |
| Q1 | 82 (14.8%) | 9 (9.0%) | 0 (0.0%) |  |
| Q2 | 109 (19.6%) | 12 (12.0%) | 5 (17.9%) |  |
| Q3 | 98 (17.7%) | 20 (20.0%) | 7 (25.0%) |  |
| Q4 | 129 (23.2%) | 20 (20.0%) | 3 (10.7%) |  |
| Q5 | 137 (24.7%) | 39 (39.0%) | 13 (46.4%) |  |

Abbreviations: NHANES, National Health and Nutrition Examination Survey; NPR, non-proliferative retinopathy; PR, proliferative retinopathy; PIR, ratio of family income to poverty; BMI, body mass index; SBP, systolic blood pressure; DBP, diastolic blood pressure; HbA1c, glycated hemoglobin; WC, waist circumference; ABSI, abdominal body shape index; WWI, weight-adjusted-waist index.

**Table S3 Association between WWI and retinopathy severity.**

| **WWI (cm/√kg)** | **Mild NPR** | **Moderate/Severe NPR** | **PR** |
| --- | --- | --- | --- |
| **Non-adjusted Model** |  |  |  |
| Continuous | 1.0 (ref.) | 1.7047 (1.2805, 2.2695) 0.0003 | 1.8870 (1.1421, 3.1179) 0.0132 |
| Quartile of WWI |  |  |  |
| Factor (WWI.Q5)1 | 1.0 (ref.) | 1.4361 (0.6675, 3.0898) 0.3545 | 7.6872 (0.9312, 63.4616) 0.0583 |
| Factor (WWI.Q5)2 | 1.0 (ref.) | 1.4939 (0.7004, 3.1866) 0.2990 | 5.3950 (0.6209, 46.8791) 0.1265 |
| Factor (WWI.Q5)3 | 1.0 (ref.) | 1.7048 (0.8102, 3.5871) 0.1599 | 5.5404 (0.6375, 48.1532) 0.1207 |
| Factor (WWI.Q5)4 | 1.0 (ref.) | 3.1871 (1.5858, 6.4052) 0.0011 | 12.9471 (1.6289, 102.9109) 0.0155 |
| **Adjust I** |  |  |  |
| Continuous | 1.0 (ref.) | 2.1220 (1.5111, 2.9798) <0.0001 | 1.9104 (1.0524, 3.4680) 0.0334 |
| Quartile of WWI |  |  |  |
| Factor (WWI.Q5)1 | 1.0 (ref.) | 1.7964 (0.8164, 3.9529) 0.1455 | 8.3845 (1.0004, 70.2735) 0.0500 |
| Factor (WWI.Q5)2 | 1.0 (ref.) | 1.8151 (0.8236, 4.0004) 0.1393 | 5.1877 (0.5805, 46.3588) 0.1407 |
| Factor (WWI.Q5)3 | 1.0 (ref.) | 2.4441 (1.1067, 5.3975) 0.0270 | 5.7924 (0.6405, 52.3798) 0.1179 |
| Factor (WWI.Q5)4 | 1.0 (ref.) | 4.8269 (2.1703, 10.7352) 0.0001 | 12.3588 (1.4166, 107.8202) 0.0229 |
| **Adjust II** |  |  |  |
| Continuous | 1.0 (ref.) | 2.1260 (1.4945, 3.0243) <0.0001 | 1.9176 (1.4054, 2.6166) <0.0001 |
| Quartile of WWI |  |  |  |
| (Intercept) | 1.0 (ref.) | 0.0760 (0.0086, 0.6716) 0.0204 | 0.0038 (0.0001, 0.2559) 0.0095 |
| Factor (WWI.Q5)1 | 1.0 (ref.) | 1.7564 (0.7904, 3.9032) 0.1668 | 8.2391 (0.9738, 69.7064) 0.0529 |
| Factor (WWI.Q5)2 | 1.0 (ref.) | 1.8429 (0.8250, 4.1167) 0.1360 | 4.7085 (0.5209, 42.5639) 0.1678 |
| Factor (WWI.Q5)3 | 1.0 (ref.) | 2.3273 (1.0368, 5.2238) 0.0406 | 5.5798 (0.6013, 51.7798) 0.1304 |
| Factor (WWI.Q5)4 | 1.0 (ref.) | 4.6671 (2.0453, 10.6496) 0.0003 | 11.7452 (1.3133, 105.0408) 0.0275 |

Note: Unsensitive analysis, converting the weight adjusted waist circumference index from a continuous variable to a categorical variable (quintile).

Non-adjusted model adjust for: None

Adjust I model adjust for: Gender; Age; Race

Adjust II model adjust for: Gender; Age; Race; Education; PIR; SBP; TCHOL; HDL.

Abbreviations: WWI, weight-adjusted-waist index; NPR, non-proliferative retinopathy; PR, proliferative retinopathy; PIR, ratio of family income to poverty; SBP, systolic blood pressure; TCHOL, total cholesterol; HDL, high-density lipoprotein.

**Table S4 The correlation between independent variable (WWI), dependent variable (retinopathy), and mediator variable (HbA1c)**

| **Associations between HbA1c and retinopathy** | | | |
| --- | --- | --- | --- |
| Exposure | Non-adjusted  [OR (95% CI) P-value] | Adjust I  [OR (95% CI) P-value] | Adjust II  [OR (95% CI) P-value] |
| HbA1c | 1.84 (1.73, 1.97) <0.0001 | 1.78 (1.67, 1.90) <0.0001 | 1.76 (1.65, 1.88) <0.0001 |
| **Associations between WWI and HbA1c** | | | |
| Exposure | Non-adjusted  [β (95% CI) P-value] | Adjust I  [β (95% CI) P-value] | Adjust II  [β (95% CI) P-value] |
| WWI | 0.30 (0.26, 0.34) <0.0001 | 0.30 (0.26, 0.34) <0.0001 | 0.23 (0.19, 0.27) <0.0001 |

Non-adjusted model adjust for: None

Adjust I model adjust for: Gender; Age; Race

Adjust II model adjust for: Gender; Age; Race; Education; PIR; SBP; TCHOL; HDL.

Abbreviations: WWI, weight-adjusted-waist index; HbA1c, glycated hemoglobin; PIR, ratio of family income to poverty; SBP, systolic blood pressure; TCHOL, total cholesterol; HDL, high-density lipoprotein.

**Table S5 Comparison of the ability of different obesity-related indices to predict retinopathy.**

| **Test** | **AUC (95% CI)** | | **Specificity** | | **Sensitivity** | **Accuracy** | **N-for-diagnose** | ***P* value** | **PPV** | **NPV** | **TP** | **FP** | **FN** | **TN** | ***P* value (compare with WWI)** |
| --- | --- | --- | --- | --- | --- | --- | --- | --- | --- | --- | --- | --- | --- | --- | --- |
| **Total** |  | |  | |  |  |  |  |  |  |  |  |  |  |  |
| WWI | **0.579** (0.557, 0.600) | | 0.669 | | 0.452 | 0.642 | 8.260 | <0.001 | 0.160 | 0.897 | 309 | 1620 | 374 | 3269 |  |
| Weight | 0.537 (0.512, 0.559) | | 0.404 | | 0.662 | 0.436 | 15.114 | 0.003 | 0.134 | 0.895 | 452 | 2912 | 231 | 1977 | **0.004** |
| WC | 0.563 (0.540, 0.589) | | 0.503 | | 0.606 | 0.516 | 9.165 | <0.001 | 0.146 | 0.901 | 414 | 2430 | 269 | 2459 | 0.196 |
| BMI | 0.553 (0.532, 0.575) | | 0.378 | | 0.709 | 0.418 | 11.598 | <0.001 | 0.137 | 0.903 | 484 | 3043 | 199 | 1846 | **0.042** |
| ABSI | 0.552 (0.529, 0.574) | | 0.588 | | 0.499 | 0.577 | 11.479 | <0.001 | 0.145 | 0.894 | 341 | 2015 | 342 | 2874 | **0.002** |
| **Male** |  |  |  |  |  |  |  |  | | | | | | | |
| WWI | **0.549** (0.519, 0.577) | | 0.784 | | 0.309 | 0.720 | 10.779 | 0.002 | 0.182 | 0.879 | 116 | 521 | 260 | 1894 |  |
| Weight | 0.469 (0.439, 0.499) | | 0.489 | | 0.524 | 0.494 | 77.142 | 0.249 | 0.138 | 0.868 | 197 | 1234 | 179 | 1181 | **0.001** |
| WC | 0.547 (0.517, 0.576) | | 0.183 | | 0.888 | 0.278 | 14.103 | 0.017 | 0.145 | 0.913 | 334 | 1974 | 42 | 441 | 0.848 |
| BMI | 0.546 (0.516, 0.574) | | 0.298 | | 0.793 | 0.364 | 11.077 | 0.021 | 0.150 | 0.902 | 298 | 1696 | 78 | 719 | 0.847 |
| ABSI | 0.522 (0.492, 0.550) | | 0.485 | | 0.572 | 0.497 | 17.638 | 0.170 | 0.147 | 0.879 | 215 | 1244 | 161 | 1171 | **0.002** |
| **Female** |  |  |  |  |  |  |  |  | | | | | | | |
| WWI | **0.615** (0.585, 0.650) | | 0.629 | | 0.547 | 0.620 | 5.676 | <0.001 | 0.155 | 0.918 | 168 | 918 | 139 | 1556 |  |
| Weight | 0.535 (0.500, 0.568) | | 0.720 | | 0.362 | 0.680 | 12.277 | 0.033 | 0.138 | 0.901 | 111 | 693 | 196 | 1781 | **<0.001** |
| WC | 0.576 (0.542, 0.612) | | 0.562 | | 0.593 | 0.566 | 6.448 | <0.001 | 0.144 | 0.918 | 182 | 1083 | 125 | 1391 | **0.009** |
| BMI | 0.565 (0.534, 0.599) | | 0549 | | 0.593 | 0.554 | 7.035 | <0.001 | 0.140 | 0.916 | 182 | 1115 | 125 | 1359 | **0.006** |
| ABSI | 0.566 (0.533, 0.598) | | 0.682 | | 0.420 | 0.653 | 9.835 | <0.001 | 0.141 | 0.905 | 129 | 788 | 178 | 1686 | **<0.001** |

Abbreviations: AUC, area under the curve; CI, confidence interval; PPV, positive predictive value; NPV, negative predictive value; TP, true positive; FP, false positive; FN, false negative; TN, true negative; WWI, weight-adjusted-waist index; WC, waist circumference; BMI, body mass index; ABSI, abdominal body shape index.

**Table S6 Comparison of the ability of WWI and different fundamental predictors to predict retinopathy.**

| **Test** | **AUC (95% CI)** | **SP** | **SE** | **Acc** | **N-for-diagnose** | ***P* value** | **PPV** | **NPV** | **TP** | **FP** | **FN** | **TN** | ***P* value**  **(compare)** |
| --- | --- | --- | --- | --- | --- | --- | --- | --- | --- | --- | --- | --- | --- |
| **Basic Model** |  |  |  |  |  |  |  |  |  |  |  |  |  |
| WWI | 0.579 (0.557, 0.600) | 0.669 | 0.452 | 0.642 | 8.260 | <0.001 | 0.160 | 0.897 | 309 | 1620 | 374 | 3269 |  |
| Age | 0.568 (0.545, 0.589) | 0.480 | 0.662 | 0.503 | 7.040 | <0.001 | 0.151 | 0.910 | 452 | 2541 | 231 | 2348 | 0.433 |
| Gender | 0.528 (0.508, 0.548) | 0.506 | 0.551 | 0.512 | 17.685 | 0.006 | 0.135 | 0.890 | 376 | 2415 | 307 | 2474 | 0.004 |
| Race | 0.581 (0.560, 0.603) | 0.585 | 0.558 | 0.582 | 7.002 | <0.001 | 0.158 | 0.905 | 381 | 2029 | 302 | 2860 | 0.775 |
| Education | 0.582 (0.560, 0.603) | 0.474 | 0.640 | 0.494 | 8.823 | <0.001 | 0.145 | 0.904 | 437 | 2574 | 246 | 2315 | 0.727 |
| PIR | 0.559 (0.537, 0.581) | 0.482 | 0.627 | 0.500 | 9.195 | <0.001 | 0.145 | 0.902 | 428 | 2532 | 255 | 2357 | 0.225 |
| **Joint Model (compare with basic model)** | | | | | | | | | | | | | |
| Age-WWI | 0.591 (0.569, 0.613) | 0.376 | 0.769 | 0.424 | 6.915 | <0.001 | 0.147 | 0.921 | 525 | 3051 | 158 | 1838 | 0.016 |
| Gender-WWI | 0.589 (0.567, 0.611) | 0.382 | 0.750 | 0.427 | 7.592 | <0.001 | 0.145 | 0.916 | 512 | 3021 | 171 | 1868 | <0.001 |
| Race-WWI | 0.623 (0.601, 0.646) | 0.628 | 0.561 | 0.619 | 5.311 | <0.001 | 0.174 | 0.911 | 383 | 1821 | 300 | 3068 | <0.001 |
| Education-WWI | 0.605 (0.583, 0.627) | 0.590 | 0.564 | 0.587 | 6.502 | <0.001 | 0.161 | 0.906 | 385 | 2004 | 298 | 2885 | 0.002 |
| PIR-WWI | 0.592 (0.570, 0.614) | 0.472 | 0.672 | 0.497 | 6.939 | <0.001 | 0.151 | 0.912 | 459 | 2581 | 224 | 2308 | <0.001 |

Abbreviations: WWI, weight-adjusted-waist index; AUC, area under the curve; CI, confidence interval; SP, specificity; SE, sensitivity; Acc, accuracy; PPV, positive predictive value; NPV, negative predictive value; TP, true positive; FP, false positive; FN, false negative; TN, true negative; PIR, ratio of family income to poverty.


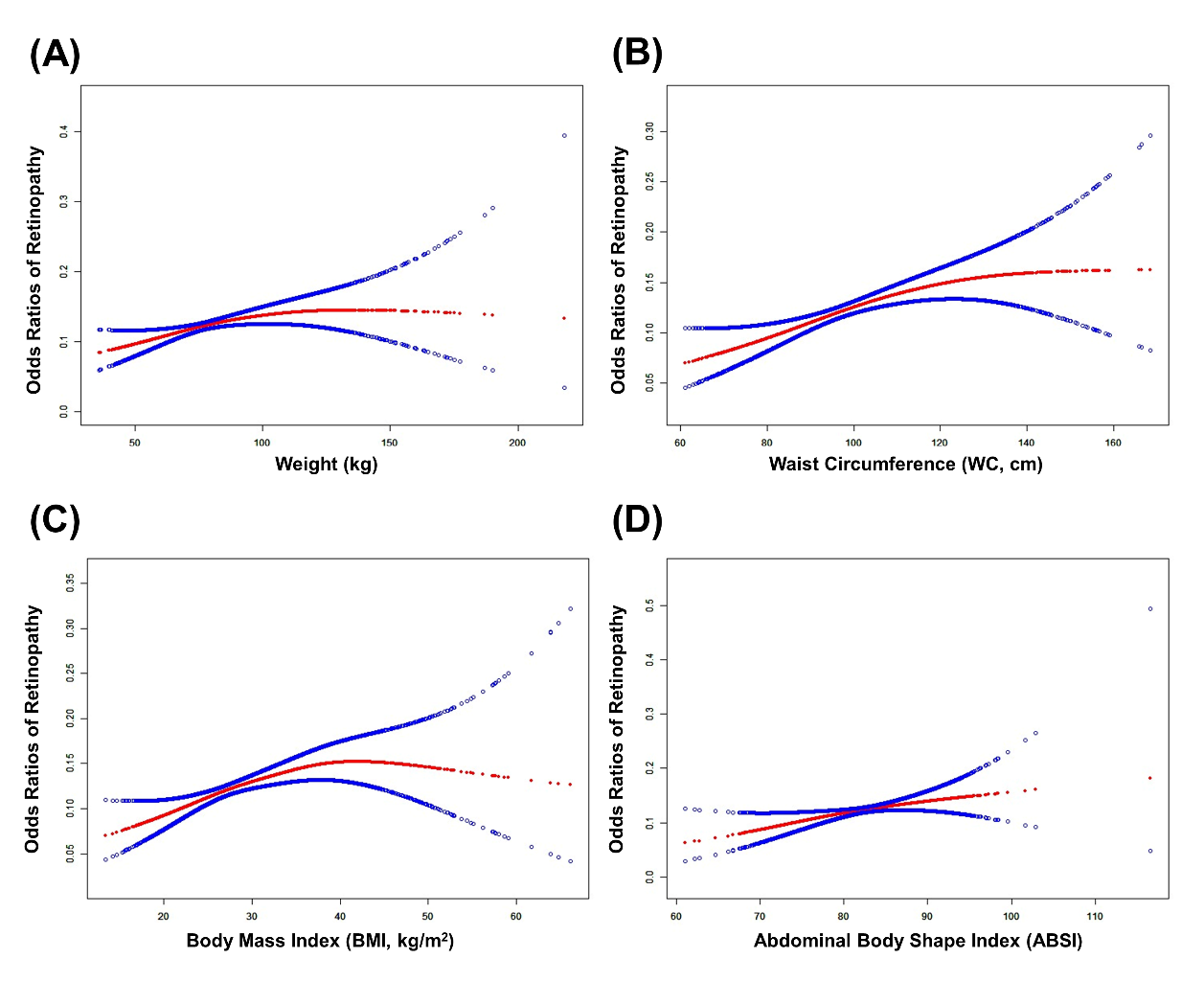


**Figure S1 Smooth curve fitting for weight, WC, BMI, and ABSI with retinopathy risk.**

(A) A positive correlation is observed between body weight and retinopathy risk, characterized by an overall linear trend with a gradual increase in risk; (B) WC is positively associated with retinopathy risk, with a more pronounced upward trend in higher ranges of WC; (C) BMI is positively correlated with retinopathy risk, but the overall trend is relatively flat compared to other measures; (D) While ABSI shows a non-linear relationship with retinopathy risk, with a sharp risk increase at higher values, it remains less interpretable in clinical settings.

Abbreviations: WWI, weight-adjusted-waist index; WC, waist circumference; BMI, body mass index; ABSI, abdominal body shape index.
